# Out-of-Hospital Cardiac Arrest due to Drowning in North America: Comparison of Patient Characteristics between Survival and Mortality Groups

**DOI:** 10.1155/2020/9193061

**Published:** 2020-10-17

**Authors:** Zhenhua Huang, Wanwan Zhang, Jinli Liao, Zhihao Liu, Yan Xiong, Hong Zhan

**Affiliations:** The Division of Emergency Medicine, The First Affiliated Hospital of Sun Yat-sen University, No. 58 Zhongshan 2th Road, Yuexiu District, Guangzhou 510080, China

## Abstract

Out-of-hospital cardiac arrest (OHCA) due to drowning carries high morbidity and mortality. There are a few studies on drowning-related out-of-hospital cardiac arrest (OHCA), in which patients are followed from the scene to hospital discharge. This study aims to compare patient characteristics between the survival group and mortality group of OHCA due to drowning. OHCA due to drowning cases were selected from the North America Termination of Resuscitation Association database between 2011 and 2015. The retrospective analysis of epidemiological characteristics and clinical features of all OHCA patients were performed. Of the 17,094 OHCA cases in the registry, 54 cases of OHCA due to drowning were included in this study. Among the 54 OHCAs due to drowning, 7 (13.0%) survived, while 47 (87.0%) died. Compared to the mortality group, the survival group had a higher bystander witness rate (57.1% versus 17.0%, *p* < 0.05), higher asystole rate (42.9% versus 78.7%, *p* < 0.05), and higher mild therapeutic hypothermia rate (28.6% versus 2.1%, *p* < 0.05). In addition, a large proportion of survivors were children (71.4%) and males (71.4%). Survival among OHCA's due to drowning was found to be improved with a higher bystander rate, higher asystole rate, and higher mild hypothermia rate. In addition, children and males comprised the majority of survivors.

## 1. Introduction

Drowning is a major global public health concern with high morbidity and mortality, resulting in significant healthcare-related, societal, and financial burdens [1–3]. In 2004, at least 382,000 people documented by the World Health Organization (WHO) died because of drowning. Adult males comprised the largest mortality group due to drowning [4]. Out-of-hospital cardiac arrest (OHCA) due to drowning mostly occurred in prehospital circumstances, and OHCA due to drowning comprised almost 0.5%–1.0% of total OHCA [5].

However, at present, there is a limited research on OHCA due to drowning around the world. Besides some case reports, there is limited systematic analysis of patient characteristics, e.g., between the survival group and mortality group. Therefore, this study aims to compare the patient characteristics between the survival group and mortality group from OHCA due to drowning cases included in the North American Termination of Resuscitation studies between 2011 and 2015.

## 2. Method

### 2.1. Study Population

The OHCA due to drowning cases were selected from the North American Termination of Resuscitation Studies data between 2011 and 2015 (total of 17,094 OHCA cases), covering data from the United States and Canada.

### 2.2. Inclusion and Exclusion Criteria

The inclusion criteria were patients who experienced OHCA due to drowning with clear outcomes from the North American Termination of Resuscitation Studies from 2011 to 2015. The exclusion criteria were cases without the emergency medical services (EMS) record, cases with patients who have signed do not resuscitate (DNR) orders, OHCA with nondrowning etiology, and cases without clear outcomes.

### 2.3. Data Processing

Included cases were divided into the survival group and death group according to hospital discharge outcome. The following patient characteristics were identified: age, gender, witness status (EMS or bystander witness), bystander cardiopulmonary resuscitation (yes or no), location of cardiac arrest (private or public), EMS response time (interval from call for ambulance until ambulance arrival), adrenaline dosage (large dosage was defined as ≥3 mg), application of prehospital advanced airway management, return of spontaneous circulation (ROSC) in prehospital environment (yes or no), and survival at discharge (yes or no).

### 2.4. Statistical Analysis

All statistical calculations were performed using the statistical program SPSS 20.0 (IBM Inc., Armonk, NY, USA). The quantitative variables are expressed as the mean ± standard deviation (SD), while the qualitative variables are expressed as the absolute value and percentage. The independent sample *t*-test was used in terms of the comparison of quantitative variables between groups. The continuous variables were nonnormal distribution, and the four digit (IQR) test was used in the aspects of the quantitative variables, while the chi-square test was used in the comparison of the qualitative variables. All of the above statistical tests were double-sided tests, which were considered statistically significant in *p* < 0.05.

## 3. Results

### 3.1. Patient Characteristics

**Figure 1:**
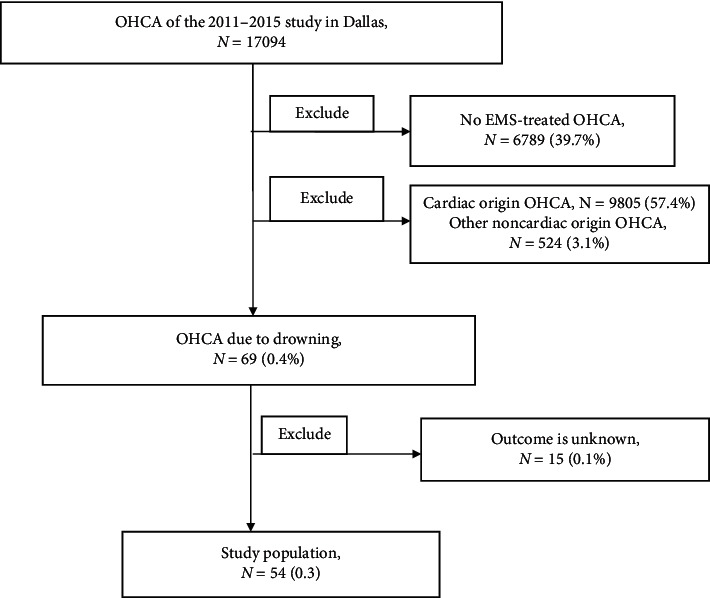
Target cohort and exclusions. OHCA indicates out-of-hospital cardiac arrest; Epistry, Epidemiological Cardiac Arrest Registry; PRIMED, prehospital resuscitation using an impedance valve and early versus delayed analysis; DFW, Dallas–Fort Worth; ROC, Resuscitation Outcomes Consortium; ROSC, return of spontaneous circulation; (*N*), number.

Out of the 17,094 OHCA cases from the database, there were 69 OHCA due to drowning, of which 15 were excluded for unknown outcomes, leaving us 54 cases (Figure 1). Of these, 43 (79.6%) cases were males, while the rest were females, and the median age was 30 (IQR 8.5–51.5). The specific epidemiological characteristics of patients with OHCA due to drowning are shown in Table 1. As shown in Table 1, the number of EMS witnessed was 0, and bystander witnessed was 12 (22.2%). Bystander CPR was performed in 26 cases (48.1%). The median EMS response time was 4.7 (3.4–5.5) minutes, and the duration of CPR was 20.1 (14.8–26.8) minutes. Application of prehospital advanced airway was accomplished in 22 (40.7%) patients. The usage rate of adrenaline was 83.3%. There were 5 patients with prehospital ROSC and 3 patients with mild therapeutic hypothermia (MTH). Among the 54 drowning OHCAs, compared to the death group, within the survival group, there was a higher instance of bystander witnessed rate (57.1% versus 17.0%, *p* < 0.05), lower asystole rate (42.9% versus 78.7%, *p* < 0.05), shorter duration time of cardiopulmonary resuscitation (CPR) (10.5 min versus 22.8 min, *p* < 0.05), and higher mild therapeutic hypothermia rate (28.6% versus 2.1%, *p* < 0.05).

### 3.2. Clinical Characteristics of Survivors after Hospital Discharge

As shown in Table 2, among all of the OHCA due to drowning, only 7 (13.0%) survived to hospital discharge, and the large proportion of survivors were children (71.4%) and males (71.4%). Most of the submersion incidents occurred in uptown (71.4%), and average EMS response time was 3.7 minutes. Among the survivors, 4 (57.1%) cases were witnessed by bystanders, and the initial first recognized rhythms were ventricular fibrillation (VF)/ventricular tachycardia (VT), pulseless electrical activity (PEA), and asystole. Furthermore, 4 (57.1%) patients had prehospital ROSC, and CPR was performed by bystanders in the 4 patients with CPR duration time of 11.9 min. Advanced airway management was accomplished in 2 (28.6%) patients. With a long hospitalization time, survivors had a good neurological outcome indicated and were assessed according to the cerebral performance categories. The use of epinephrine did not appear to affect the outcome of OHCA due to drowning (data deficiencies).

## 4. Discussion

### 4.1. Characteristics of the Drowned OHCA

These were the few reports available to evaluate the OHCA characteristics of drowning in North America. There were 17,094 OHCAs in North America from OHCA Registry between 2011 and 2015, of which 69 (0.4%) were drowned OHCA. In this investigation, the amount of included patients with OHCA due to drowning were only 54 (0.3%). The mortality was as much as 87%, with the hospital discharge survival rate of 13%.

Of these survivors, we found that patients were younger, and it appeared that children were more prone to survive drowning. Previous studies have also showed that children suffering from OHCAs had better outcomes than adults [6–8]. Previous studies also found higher survival and discharge rates when the downing was bystander witnessed [4,6]. In the survival group, the probability of occurrence in living residence was higher (71.4%), 4 (57.1%) patients were witnessed by bystanders, and bystander CPR was commenced in 4 (57.1%) cases with the average CPR duration time of 11.9 minutes. Drowning occurrences in the homes were more likely to have family members or friends nearby, which can account for its high witness rate. When directly witnessed, calling for medical help and early bystander CPR was much more prompt, which can explain the mean EMS time of 3.7 minutes in the survival group. Furthermore, 4 (57.1%) patients had prehospital return of spontaneous circulation (ROSC), and the advanced air way managements were accomplished in 3 (42.9%) patients after the medical intervention was carried out.

### 4.2. Characteristics of Survival Outcomes

Our study found that children are more likely to survive from drowning, as found in other previous studies [6,9]. This is possibly explained by the advantages associated with children, who are generally younger, healthier, with hearts not yet plagued by coronary heart disease compared to the elderly.

#### 4.2.1. Witness Status

The survey of Dyson et al. [4] demonstrates that the survival discharge rate in patients with OHCA caused by drowning in Australia is 7.8%, which is less than the 13% in our study. The witness rate in the survey of the Kylie Dyson study is only 17.9%, while it is 57.1% in our study. This supports our study in which a high witness rate is associated with a significantly increased survival rate.

#### 4.2.2. Advanced Air Way Management

There is little research or information on the effect of the advanced air way management connected to OHCA due to drowning. As known, the immediate cause of cardiac arrest due to drowning is hypoxia caused by dyspnea. The advanced air way managements, such as a trachea cannula and tracheotomy, can directly correct the hypoxia situation of the body. In this study, the advanced air way management was carried out in 22 cases. Of these cases, 3 patients survived to hospital discharge. The advanced air way management rate in both groups did not differ significantly (42.9% versus 40.4, *p* > 0.05), but because of the lack of samples available of patients with advanced air way management performed, it is unclear whether the correction of hypoxia of the body is significantly related to the survival discharge rate among patients with OHCA due to drowning. We need more data illustrating the relationship between prompt advanced air way management and survival rate after hospital discharge to make any further conclusions.

#### 4.2.3. Presence of Shockable Rhythm

The initial ECG rhythm in patients with OHCA due to hypoxia caused by drowning is most often asystole. Compared to the survival group, there are higher asystole rates (78.7% versus 42.9%, *p* < 0.05) in the death group. With the presence of nonshockable rhythm (asystole), it is difficult to reverse the heart rhythm to sustain stable hemodynamics. The research of Zheng et al. [10] in 2016 displayed that conversion to shockable rhythms is associated with better outcomes in out-of-hospital cardiac arrest patients with initial asystole but not in those with pulseless electrical activity. We have a similar finding in this study.

#### 4.2.4. Mild Therapeutic Hypothermia

The previous study of Reinikainen M in 2012 demonstrated that the early use of mild therapeutic hypothermia treatment in OHCA can improve the survival rate [11]. As shown in Table 2, there are higher mild therapeutic hypothermia rates (28.6% versus 2.1%, *p* < 0.05) in the survival group as compared to the death group. To make a conclusion that the early use of mild therapeutic hypothermia treatment in OHCA due to drowning is connected to a significantly increased survival rate, we need to collect more data. As of now, its relationship to better outcomes is still unclear.

## 5. Limitations

Our study has several limitations.

  First, as a retrospective observational study, the present analysis may be the subject to biases caused by unmeasured confounding factors. Therefore, our results need to be interpreted with caution.
  Second, the patients with OHCA caused by drowning included in our study were only 54. Due to the limited data, this study can only conduct a descriptive analysis. We cannot study which factors are favorable for survival and which are harmful by using the logistic regression model or other statistical models. But we believe that this study is meaningful because we know little about drowning-related OHCA, and we insist more studies should be conducted in the future.

## 6. Conclusion

The survival rate of OHCA caused by different etiologies is not obviously different. Important predictors of survival are age and witnessed status. With the presence of advanced air way management and the early use of mild therapeutic hypothermia in OHCA due to drowning, the survival rate will likely be higher.

## Figures and Tables

**Table 1:** Comparison of epidemiological characteristics of out-of-hospital cardiac arrest due to drowning.

Demographics and characteristics	Total, *N* = 54	Discharged alive, *N* = 7 (13.0)	Died, 47 (87.0)	*p*
Baseline characteristics				
Age (years), median (IQR)	30 (8.5–51.5)	9.5 (2.8–48.8)	30 (10–53)	0.235
Male, *N* (%)	43 (79.6)	5 (71.4)	38 (80.9)	0.621
Public place, *N*(%)	18 (33.3)	2 (28.6)	16 (34.0)	1.000
Event characteristics	—	—	—	—

Witnessed status				
EMS-witnessed, *N* (%)	**0**	**0**	**0**	—
Bystander-witnessed, *N* (%)	12 (22.2)	4 (57.1)	8 (17.0)	0.036
Bystander CPR, *N* (%)	26 (48.1)	3 (42.9)	23 (48.9)	1.000
AED shock delivered, *N* (%)	0	0	0	—

Initial ECG rhythm				
VF/VT, *N* (%)	3 (5.6)	2 (28.6)	1 (2.1)	0.046
PEA, *N* (%)	7 (13.0)	1 (14.3)	6 (12.8)	1.000
Asystole, *N* (%)	40 (74.1)	3 (42.9)	37 (78.7)	0.031
Perfusing, *N* (%)	0	0	0	—
AED—no shock advised *N* (%)	1 (1.9)	1 (14.3)	0	—

EMS interventions				
911 call—EMS arrival (min), median (IQR)	4.7 (3.4–5.5)	3.5 (3.0–4.7)	4.8 (3.5–5.7)	0.067
CPR duration (min), median (IQR)	20.1 (14.8–26.8)	10.5 (8.7–17.5)	22.8 (16.4–30.7)	0.003
Advanced airway attempted, *N* (%)	22 (40.7)	3 (42.9)	19 (40.4)	1.000
Epinephrine administered, *N* (%)	45 (83.3)	4 (57.1)	41 (87.2)	0.081
Dose epinephrine (mg), median	3 (1–4)	1.1 (0.1–3.5)	0.137	0.137
EMS shock delivered, *N* (%)	8 (14.8)	2 (28.2)	6 (12.8)	0.291
Prehospital ROSC	5 (9.3)	4 (57.1)	1 (2.1)	—
Therapeutic hypothermia, *N* (%)	3 (5.6)	2 (28.6)	1 (2.1)	0.041

IQR, interquartile range; EMS, emergency medical services; VF, ventricular fibrillation; VT, ventricular tachycardia; PEA, pulseless electrical activity; AED, automated external defibrillator; CPR, cardiopulmonary resuscitation; ICU, intensive care unit.; DNR, do not resuscitate.

**Table 2:** The clinical characteristics of patients who survived to hospital discharge.

	Age	Sex	First known rhythm	Place of CA	Bystander witnessed	Bystander CPR	Response time (min)	Advanced airway	Dose of epinephrine (mg)	CPR duration (min)	Days in CCU	MRS
1	11	Male	VF/VT	Place of recreation	Yes	No	4.7	No	Unknown	13.1	Unknown	Unknown
2	2	Male	PEA	Home residence	No	No	3.0	No	0	10.2	2	0
3	51	Male	Asystole	Home residence	No	No	5.4	Yes	4	20	25	0
4	3	Female	AED no shock	Home residence	Yes	Yes	3.1	No	Unknown	3.2	12	4
5	Unknown	Male	VF/VT	Place of recreation	Yes	No	3.5	Yes	Unknown	8.7	20	3
6	8	Female	Asystole	Home residence	No	Yes	3.7	No	0	10.5	31	5
7	48	Male	Asystole	Home residence	Yes	Yes	2.5	No	2	17.5	Unknown	Unknown

EMS, emergency medical services; VF, ventricular fibrillation.; VT, ventricular tachycardia.; PEA, pulseless electrical activity; AED, automated external defibrillator; CPR, cardiopulmonary resuscitation; ICU, intensive care unit; DNR, do not resuscitate; ROSC, return of spontaneous circulation; ROSC, return of spontaneous circulation.

## Data Availability

The data used to support the findings of this study are available from the corresponding author upon request.
